# Evaluation of users’ level of satisfaction for an artificial intelligence-based diagnostic program in pediatric rare genetic diseases

**DOI:** 10.1097/MD.0000000000029424

**Published:** 2022-07-15

**Authors:** In Hee Choi, Go Hun Seo, JeongYun Park, Yoon-Myung Kim, Chong Kun Cheon, Yoo-Mi Kim, Arum Oh, Jung Hye Byeon, Eungu Kang, Young-Lim Shin, Ji Eun Lee, Su Jin Kim, Hee Joon Yu, Woo Jin Kim, Byung Yoon Choi, Bong Jik Kim, Young Ho Kim, Gi Jung Im, Hyo-Jeong Lee, Hyun Ji Kim, Se-Hee Han, Beom Hee Lee, Baik-Lin Eun

**Affiliations:** a Department of Genetic Counseling, University of Ulsan College of Medicine, Seoul, South Korea; b 3billion Inc., Seoul, South Korea; c Department of Clinical Nursing, University of Ulsan, Seoul, South Korea; d Department of Pediatrics, Gangneung Asan Hospital, College of Medicine, University of Ulsan, Gangneung, South Korea; e Division of Medical Genetics and Metabolism, Department of Pediatrics, Pusan National University School of Medicine, Pusan National University Children’s Hospital, South Korea; f Department of Pediatrics, Pediatric Center, Chungnam National University Sejong Hospital, College of Medicine, Chungnam National University, Sejong, South Korea; g Department of Pediatrics, Chungbuk National University Hospital, Chungbuk National University College of Medicine, Cheongju, South Korea; h Department of Pediatrics, Korea University College of Medicine, Seoul, South Korea; i Department of Pediatrics, Soonchunhyang University Bucheon Hospital, Soonchunhyang University College of Medicine, Bucheon, South Korea; j Department of Pediatrics, Inha University Hospital, Inha University College of Medicine, Incheon, South Korea; k Department of Pediatrics, Hallym University Sacred Heart Hospital, Gyeonggi-do, South Korea; l EONE Laboratories, Harmony-ro, Yeonsu-gu, Incheon, South Korea; m Department of Otorhinolaryngology, Seoul National University Bundang Hospital, Seongnam, South Korea; n Department of Otolaryngology–Head and Neck Surgery, Chungnam National University College of Medicine, Chungnam National University Sejong Hospital, Sejong, South Korea; o Department of Otorhinolaryngology, Seoul National University Boramae Medical Center, Seoul National University College of Medicine, Seoul, South Korea; p Department of Otorhinolaryngology, Korea University College of Medicine, Seoul, South Korea; q Department of Otorhinolaryngology-Head and Neck Surgery, Hallym University College of Medicine, Chuncheon, South Korea; r Department of Otorhinolaryngology-Head and Neck Surgery, Inha University College of Medicine, Incheon, South Korea; s Korea University Guro Hospital, Seoul, South Korea; t Medical Genetics Center, Asan Medical Center, University of Ulsan College of Medicine, Seoul, South Korea.

**Keywords:** artificial intelligence-based diagnostic program, buccal swab, medical data analysis diagnostic intelligent software, rare disease, whole-exome sequencing

## Abstract

The artificial intelligence (AI)-based genetic diagnostic program has been applied to genome sequencing to facilitate the diagnostic process. The objective of the current study was to evaluate the experience and level of satisfaction of participants using an AI-based diagnostic program for rare pediatric genetic diseases. The patients with neurodevelopmental disorders or hearing impairments, their guardians, and their physicians from 16 tertiary general hospitals were enrolled. The study period was from April 2020 to March 2021. A survey was designed to assess their experience and level of satisfaction. A total of 30 physicians and 243 patients and guardians (199 neurodevelopmental disorders and 44 hearing impairments) completed the survey. DNA samples of the subjects were collected through buccal swabs or blood collection: 211 subjects (86.8%) through buccal swab and 29 subjects (11.9%) through blood collection. Average turnaround time for result receipt was 57.54 ± 32.42 days. For the sampling method, 193 patients and guardians (81.1%) and 28 physicians (93.3%) preferred buccal swab. The level of satisfaction of the 2 groups participating in the AI-based diagnostic program was 8.31 ± 1.71 out of 10 in the patient and guardian group and 8.42 ± 1.23 in the physician group. Clinicians, patients, and guardians are satisfied with the AI-based diagnostic program in general. With an increase in AI-based precision medicine solutions, the evaluation of the user’s satisfaction with appropriate provision will help improve personal health care.

## 1. Introduction

A rare disease is defined as a condition affecting <1 in 2000 to 200,000 individuals according to the population.^[1–3]^ The number of rare diseases is estimated to be approximately 7000 and >5000 are caused by genetic defects.^[4,5]^ In ~75% of rare diseases, the symptoms appear during childhood and result in various clinical symptoms, complications, and morbidity. Moreover, 30% of the patients reportedly die before reaching the age of 5 years.^[5–7]^ Therefore, its early diagnosis is important for appropriate early intervention, including prediction of prognosis, education, management, and possible surgical approaches.

Due to clinical and genetic diversity as well as the rarity of each rare disease, its genetic confirmation has been challenging. With traditional genetic tests such as chromosomal, biochemical, and gene-by-gene tests, called as “Diagnostic Odyssey,” it takes a long time to conduct a diagnosis in a suspected patient.^[7]^ However, since the early 2000s, with the advent of massively parallel sequencing or the next-generation sequencing (NGS) technique, the process and speed of genetic diagnosis have greatly improved to a diagnostic rate of 30% to 40% in patients with suspected genetic diseases.^[5,8–12]^ Genome sequencing using the NGS technique, such as whole-exome sequencing (WES) or whole-genome sequencing, showed higher clinical utility than the traditional tests.^[9,10]^ However, as a high number of variants are detected in a patient, it is challenging to categorize and interpret these variants and find a causative variant responsible for the patient’s phenotype.^[7,12–14]^

Recently, in the healthcare industry, artificial intelligence (AI) has been applied to facilitate disease diagnosis and treatment development. In particular, AI technology has improved the process of genetic diagnosis by fast variant interpretation through machine learning of the vast genomic data.^[15]^

In a previous study, our group introduced a new, streamlined, automated genetic variant prioritization system, termed EVIDENCE (3billion Inc., Seoul, South Korea). EVIDENCE analyses and prioritizes over 100,000 genetic variants according to a patient’s phenotype within minutes.^[16]^ This software showed a comparable diagnostic rate in various spectrums of genetic diseases as in a single-center study with the previously reported studies.^[16]^

In the current study, we applied this EVIDENCE system to the genetic diagnosis process of delayed neurodevelopment and hearing impairments in multiple hospitals in Korea and assessed the experience and level of satisfaction of patients, their guardians, and clinicians who participated in the study.

## 2. Methods

### 2.1. Study design and participants

This study was an observational study to evaluate the experience of using EVIDENCE by patients with rare pediatric diseases, their guardians, and physicians at tertiary hospitals in Korea. The study period was from April 2020 to March 2021. The sample size was not calculated using statistical analysis. Based on the research budget, we recruited as many patients as possible.

AI-based diagnostic program was applied to all patients with neurodevelopmental disorders and hearing impairments who visited the hospital. Neurodevelopmental disorders refer to those pertaining to delayed bodily gross motor, social skills, language, cognition, and fine motor. The inclusion criteria for the patients with neurodevelopmental disorders were patients aged >5 months with neurodevelopmental delay, documented by the Korean infant and child development test,^[17]^ the genetic defect was strongly suspected by the physician, and no pathogenic variant was identified using single-gene tests or chromosomal assay.

The inclusion criteria for selecting patients with hearing impairment were moderate hearing loss with an average of >25 dB at 500, 1000, 2000, and 4000 Hz in pure-tone audiometry,^[18]^ and the patients with conduction deafness and asymmetric hearing background were excluded.

Informed consent was obtained from all participants included in the study. The researcher explained the purpose of the study in-person to the patients, guardians, or physicians who participated in the AI-based genetic diagnostic program and completed the questionnaire after obtaining voluntary written consent. For participants under 18 years of age or those who had cognitive impairment or intellectual disability, the questionnaire was completed after obtaining written consent from the participant’s legal representative. For ethical protection of the human subjects, approval was obtained from the institutional review board at each of the participating hospitals (approval numbers: 2020-05-010, 04-2020-012, 2020-03-096, 2020-03-031-001, 2020AN0332, 2020AS0186, 2020-05-041-003, 2020-05-040-001, 2020-06-030-002, 2020-L06-01, B-2008/631-405, 2020-04-032, 10-2020-040, 2020AN0303, 2020-06-030-002, and 2020-04-051-002).

### 2.2. Study tools

For the evaluation of the AI-based genetic diagnostic program, the researcher developed 2 questionnaires with 15 and 16 questions for the patient and guardian groups and the physician group, respectively, through literature review. We received expert advice and secured the reliability of the questionnaire items.

#### 2.2.1. Patient and guardian group.

In the patient and guardian group, data on the relationship with the patient, age, and history of visiting other hospitals for medical examination were collected as general characteristics. For specific characteristics associated with the AI-based diagnostic program, the objective for participating, sample collection method, preferred test method, and the reason for the preference were investigated. Reliability of the diagnosis and test result, turnaround time (TAT), understanding of the test result, and level of benefits in diagnosis and treatment were assessed using the Likert scale: 1 point for “strongly disagree,” 2 points for “disagree,” 3 points for “neutral,” 4 points for “agree,” and 5 points for “strongly agree.” The overall satisfaction was measured between the score of 0 to 10 points, with a higher score indicating higher satisfaction from program participation. The reliability of the scale including all the questions was Cronbach α = 0.831.

#### 2.2.2. Physician group.

In the physician group, data on general characteristics such as specialized field and length of clinical experience were collected. For specific characteristics associated with the AI-based diagnostic program, sample collection method, preferred test method, and the reason for the preference were investigated. The convenience of the test process, adequacy of the TAT, consistency between the reported results and clinical symptoms, difference from the previous diagnostic method and process, the extent to which it helped the diagnostic quality and treatment decision, and willingness to recommend the program to other physicians were evaluated using the 5-point Likert scale: 1 point for “strongly disagree,” 2 points for “disagree,” 3 points for “neutral,” 4 points for “agree,” and 5 points for “strongly disagree.” The overall satisfaction was measured between the score of 0 and 10 points, with a higher score indicating higher satisfaction from participating in the program. The reliability of the scale including all the questions was Cronbach α = 0.705.

### 2.3. Statistical analysis

The obtained data were analyzed using SPSS Win 25.0 for Windows (SPSS Inc., Chicago, IL). The characteristics of the subjects were analyzed in terms of real numbers and percentages, and mean and standard deviation. The results of participating in the program and the comparison of participants’ perceptions about the test method were analyzed in terms of mean and standard deviation. The difference in satisfaction between the patients and guardians regarding the AI-based diagnostic program was analyzed with an independent *t* test. The satisfaction of the physicians who participated in the AI-based diagnostic program was analyzed using mean and standard deviation.

## 3. Results

### 3.1. General characteristics of the participants

The general characteristics of the participants are presented in Table 1. There were 243 patients and guardians (199 patients with neurodevelopmental disorders [81.9%] and 44 patients with hearing impairments [18.1%]). Among the guardians, 186 (77.2%) were parents of the patients. Before enrollment, 78 participants (32.5%) visited 1.67 ± 1.28 other hospitals. Among the 30 physicians who participated, 16 (53.3%) were pediatricians and 6 (20.0%) otolaryngologists. Ten physicians (33.3%) had 10 to 15 years of clinical experience and 7 physicians (23.3%) had >20 years of experience.

**Table 1:** General characteristics of the participants (N = 273).

Participants	Variables	Categories	n (%) or mean ± SD
Patients and guardians (n = 243)	Disease category	Neurodevelopmental disorder	199 (81.9)
		Hearing impairment	44 (18.1)
	Relationship with the patient	Patient	42 (17.4)
		Parents	186 (77.2)
		Others	13 (5.4)
	Experience visiting other health institutions	Yes	78 (32.5)
		No	162 (67.5)
	Frequency of institutions previously visited (n = 78)		1.67 ± 1.28 (Range: 1–10)
Physicians (n = 30)	Department	Pediatrics	16 (53.3)
		Otolaryngology	6 (20.0)
		Others	8 (26.7)
	Clinical experience, yr	<5	1 (3.3)
		5–10	6 (20.0)
		10–15	10 (33.3)
		15–20	6 (20.0)
		>20	7 (23.3)

SD = standard deviation.

### 3.2. Results of the subjects

For DNA sample collection, the buccal swab was used for 211 participants (86.8%) and blood sampling for 29 participants (11.9%). The TAT was 57.54 ± 32.42 days, and a TAT of ≤30 days was most common (41.3%; 67 subjects), followed by a TAT of 31 to 60 days (30.0%; 50 subjects) and >61 days (27.8%; 45 subjects).

Regarding sample collection, 193 patients and guardians (81.1%) and 28 physicians (93.3%) preferred the buccal swab to blood sampling because of convenience and noninvasiveness, whereas 33 (13.9%) patients and guardians and 1 (3.3%) physician preferred blood sampling because “higher accuracy” or “less chance of test errors” is expected in blood sampling or they “can do other tests simultaneously.”

### 3.3. Participant satisfaction

The genetic diagnostic rate of neurodevelopmental disorder was found to be 40.1% and that of hearing impairment was 52.3%.

**Figure 1.**
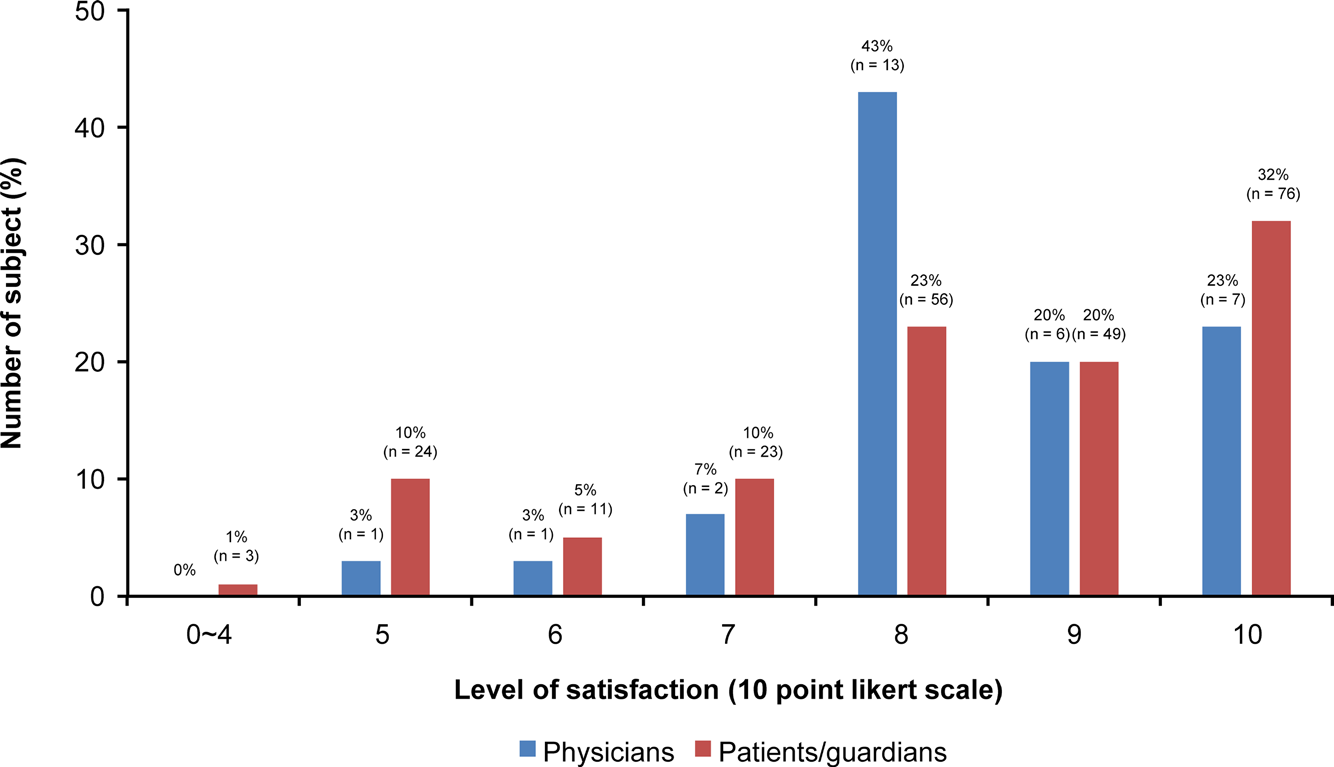
The overall satisfaction of the patients/guardians group and the physicians group.

The scores of the overall satisfaction were 8.31 ± 1.71 out of a total of 10 in the patients and guardians group and 8.42 ± 1.23 in the physicians group (Fig. 1).

**Figure 2.**
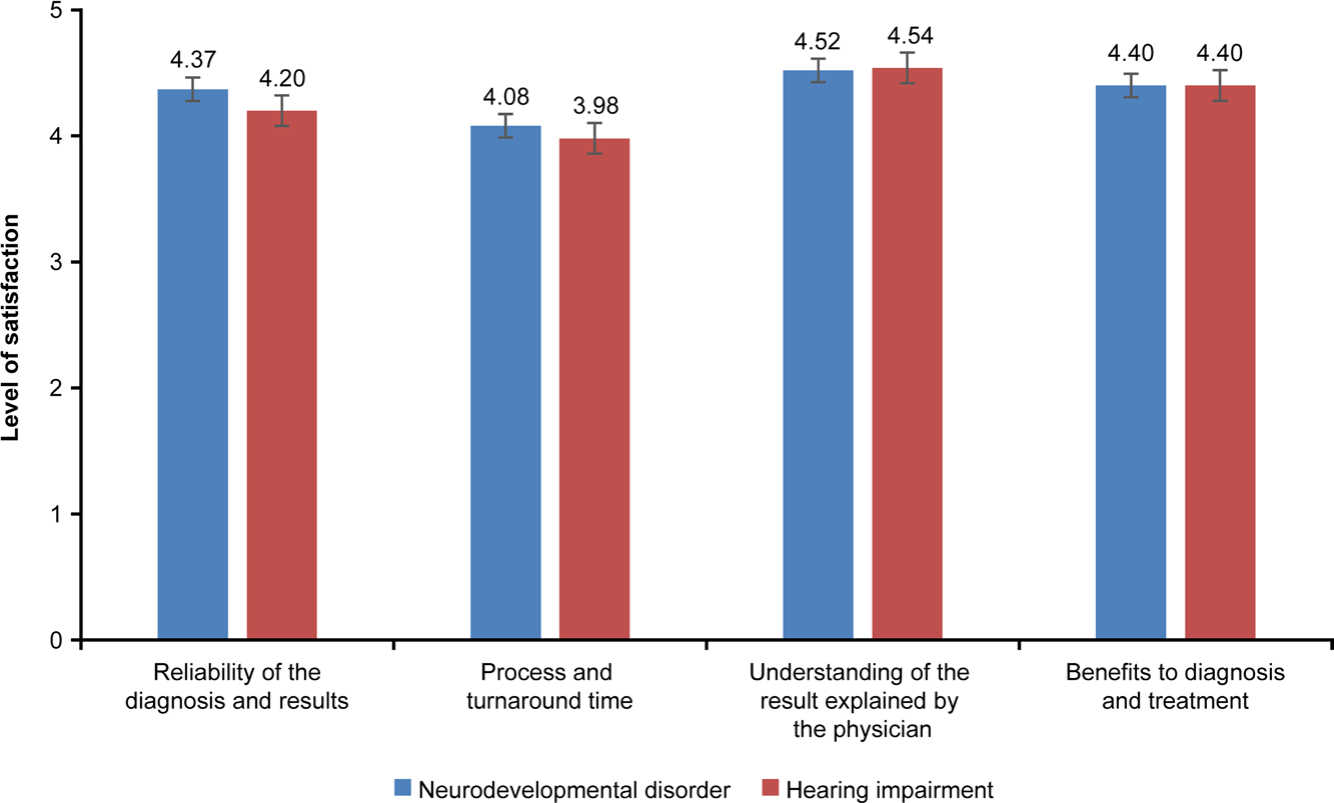
Satisfaction of the patients/guardians group.

In the patients and guardians group, the overall satisfaction was not statistically significant in 8.41 ± 1.70 of the neurodevelopmental disorder group and 7.86 ± 1.73 of the hearing impairment group (*t* = 1.910, *P* = .057; Table 2). The score for the reliability of diagnosis and test result was 4.34 ± 0.75 out of 5, test process and TAT was 4.34 ± 0.75, understanding of the test results explained by the responsible physician was 4.44 ± 0.72, and benefits of the test result was 4.37 ± 0.74 (Fig. 2). Comparing the neurodevelopmental disorder group and the hearing impairment group, there was no significant difference in the reliability of diagnosis and test result (*t* = 1.319, *P* = .188), the test process and TAT (*t* = 0.672, *P* = .502), and the benefits of the test result (*t* = 1.375, *P* = .170). Among all the items enlisted in Table 2, only understanding of the test result explained by the physician was significantly different (*t* = 3.409, *P* = .001) in both groups, with the scores in the neurodevelopmental disorder group (4.52 ± 0.68) being higher than those in the hearing impairment group (4.11 ± 0.81; Table 2).

**Table 2:** Level of satisfaction of patients and guardians (n = 243).

Items	Total	Neurodevelopmental disorder (n = 199)	Hearing impairment (n = 44)	*t (P*)
	Mean ± SD			
Overall satisfaction (1–10)	8.31 ± 1.71	8.41 ± 1.70	7.86 ± 1.73	1.910 (.057)
Reliability of the diagnosis and results	4.34 ± 0.75	4.37 ± 0.74	4.20 ± 0.76	1.319 (.188)
Process and turnaround time	4.06 ± 0.92	4.08 ± 0.91	3.98 ± 0.96	0.672 (.502)
Understanding of the result explained by the physician	4.44 ± 0.72	4.52 ± 0.68	4.11 ± 0.81	3.409 (.001)
Benefits to diagnosis and treatment	4.37 ± 0.74	4.40 ± 0.73	4.37 ± 0.74	1.375 (.170)

SD = standard deviation.

**Figure 3.**
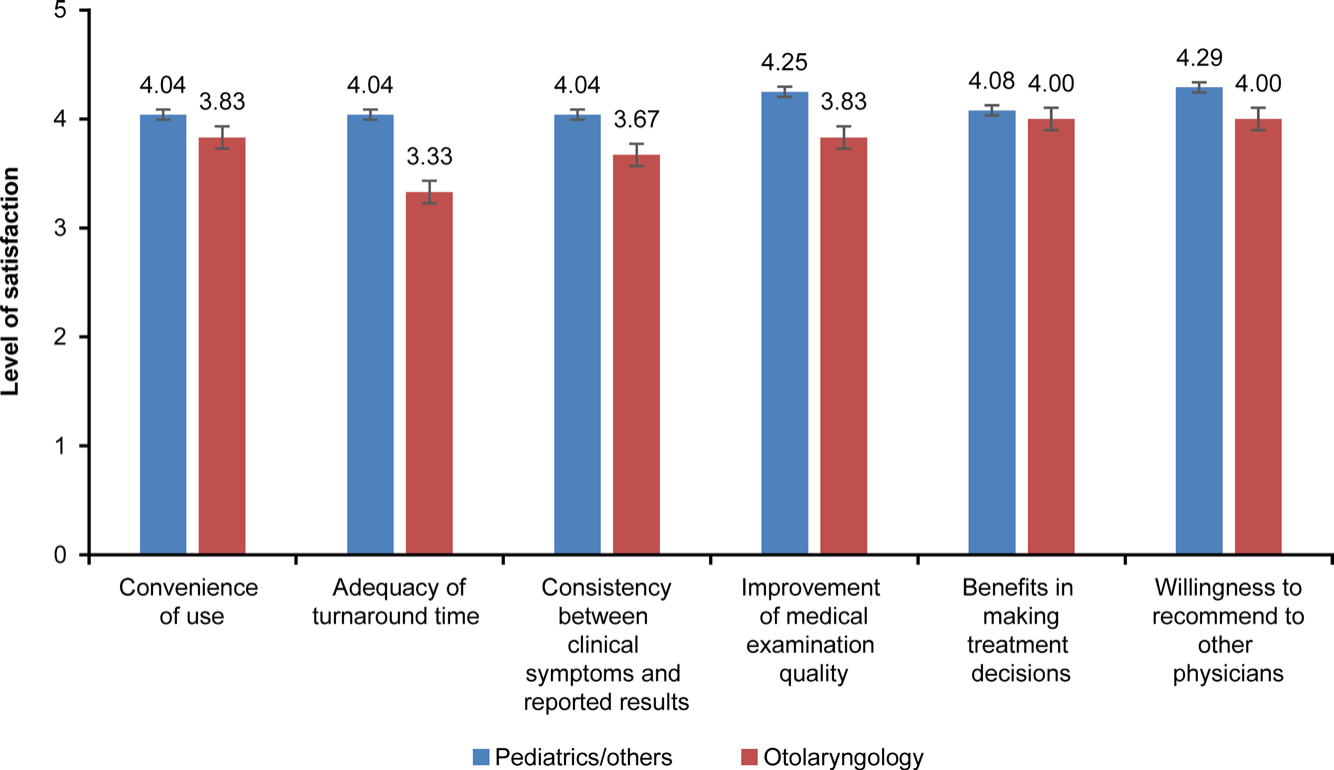
Satisfaction of the physician group.

As shown in Table 3, the overall satisfaction score of the physician group was 8.42 ± 1.23 out of 10. Although the score for the adequacy of TAT was 3.90 ± 0.72 out of 5, it was 4.0 or higher for other items (Table 3). In the physician group, the score for convenience was 4.00 ± 0.53 out of 5, adequacy of TAT was 3.90 ± 0.72, consistency between reported results and clinical symptoms was 4.00 ± 0.72, improvement of medical examination quality was 4.17 ± 0.53, benefits in making treatment decisions was 4.07 ± 0.64, and willingness to recommend to other physicians was 4.23 ± 0.57 (Fig. 3).

**Table 3:** Satisfaction of Physicians (n = 30).

**Items**	**Mean ± SD**
Overall satisfaction (1–10)	8.42 ± 1.23
Convenience of use	4.00 ± 0.53
Adequacy of turnaround time	3.90 ± 0.72
Consistency between clinical symptoms and reported results	4.00 ± 0.72
Improvement of medical examination quality	4.17 ± 0.53
Benefits in making treatment decisions	4.07 ± 0.64
Willingness to recommend to other physicians	4.23 ± 0.57

SD = standard deviation.

## 4. Discussion

This project was a Korean government-driven project to develop an AI-based medical service in South Korea, which has been named as “Dr. Answer 1.0.” It was started in 2018 to support clinical treatment and diagnosis using medical big data. It aims to provide precision medical services through early diagnosis and personalized gene treatment using genetic diagnosis. Presently, the Korean government is performing the project “Dr. Answer 2.0.” In the future, the medical AI technology will be improved to a global level based on the clinical effectiveness achieved through actual projects, and world-class medical learning data will be obtained to improve diagnosis accuracy and treatment rate of key diseases. We aimed to investigate how this program improves the diagnostic process, the provision of active or conservative treatment, and effective genetic counseling in clinical settings of rare genetic diseases.

Although AI has a beneficial effect on the diagnosis of genetic disease, the usefulness suggested by the physicians, patients, and their guardians need to be validated before we apply this technology to the general clinical practice. In the current study, the overall satisfaction of the subjects participating in the AI-based diagnostic program was found to be quite high with a mean score of 8 out of 10. Independent analysis of the 2 disease groups (Neurodevelopmental disorder and Hearing impairment) showed a higher grade of satisfaction for physicians and patients with no significant intergroup differences, suggesting the possibility that these AI-based diagnostic programs can be applied to the genetic diagnosis of various rare diseases.

One of the reasons for this high satisfaction is associated with the reliability of the diagnostic process and results both by patients and their guardians and physicians. The disease-causing genes of the subjects were not identified before they participated in the study. As a result of the AI-based diagnostic program utilizing EVIDENCE, the diagnostic rate of neurodevelopmental disorder and hearing impairment was found to be 40.1% and 52.3%, respectively. This positive rate is comparable to or higher to the previously reported WES diagnostic rate of 30% to 40%.^[5,10–12]^ The average time taken for the whole process to be completed was 57.54 ± 32.42 days; <30 days in 41.3% of cases and 31 to 60 days in 30.0% of cases, comparable to the results of other reports as approximately 40 days (range: 25–100 days).^[7,19]^ These acceptable TATs and considerable positive diagnostic rates contributed to high satisfaction. The AI-based diagnostic program showed 14% higher predictive accuracy than conventional primary AI (made by U.S. Illumina) and increased interpretation of the previously uninterpretable 11% genome regions.^[20]^

Another point to be considered is that our program helps to end “Diagnostic Odyssey” in the patients who received positive results. It has been reported that, on an average, it takes 4 to 5 years from the first appearance of rare disease symptoms to receiving a diagnosis,^[5]^ and although the process of diagnosis has become relatively faster with NGS, it still takes an average of 11 years (range: 3–42 years) from the first appearance of symptoms to receiving the WES test in a patient.^[21]^ In our patients as well, 32.5% of the participants had an experience of visiting 1.67 ± 1.28 institutions, and the maximum number was 10. By obtaining the results of diagnosis within 6 to 8 weeks, the diagnostic process has been shortened.

Sampling method is also a sensitive issue for pediatric patients. In our study, DNA samples were collected using a buccal swab in 86.8% of the participants, compared to blood sampling in 11.9%. As a noninvasive method, 81.1% of the patients and guardians and 93.3% of the physicians preferred buccal swab sampling. This noninvasive method contributed to the overall satisfaction level of the program. However, as 3% of the physicians and 11.8% of the patients and guardians preferred blood collection, test error should be considered during buccal swab sampling because of the inadequate low amount of DNA, and sometimes, resampling should be done.

The patients and guardians expressed high satisfaction toward the clinician’s explanation of the test result with an average score of 4.44 out of 5. The higher degree of satisfaction in patients with neurodevelopmental disorders than in patients with hearing impairment might be attributable in part to the fact that clinicians who treat patients with neurodevelopmental disorders are more familiar with genetic tests than those who treat patients with hearing loss and, therefore, are able to explain the results better. Considering that explaining the test result is very complex and difficult, the possibility of the positive answers being associated with the patient’s/guardian’s relationship with the clinician cannot be ruled out. It is emphasized that when delivering the results of a genetic test, the explanation should not simply include the faults or the existence of abnormality but also the information on treatment, management, and prognosis while considering the patient’s and family’s psychological, sociological, ethical, and legal status.^[22]^ The focus has been on the diagnosis of the rare disease so far, but it is more important to provide customized management for the patients and their family after the diagnosis, including the right management, social, and psychological support. For the diagnosis of pediatric rare diseases, genetic counseling for understanding the disease should be offered to the patient and the family in addition to the information on personalized management, treatment, and prenatal diagnosis.

Considering the short patient examination time in the medical health care system in South Korea, it is difficult for clinicians to provide appropriate genetic counseling before and after the test. Certified genetic counselors can explain the tests based on the disease, diagnosis, disease cause, progression, treatment, and prevention to the patient and the family and also help them make voluntary decisions by providing psychological support. However, based on the data from June 2021, the number of certified genetic counselors in Korea is only 54, which is still very low.^[23]^ The certification system began only in 2014, which is relative behind compared to United States, Europe, and other Asian countries, and the counseling fee is not recognized as medical spending. Recently, not only for rare diseases but also for other diseases with high incidence rates such as multifactorial disorders and tumors, the importance of molecular genetic diagnosis, prediction of susceptibility to disease morbidity, selecting treatment drugs, and predicting the response is increasing. Regulation to provide genetic counseling along with clinical genetic specialist consultations is required.

This is the first study evaluating the experience and level of satisfaction of an AI-based diagnostic program among clinicians, patients, and guardians. However, the present study does not encompass all rare diseases, and the obtained results are difficult to generalize. Moreover, since it was based on a self-reporting system, the answers could have been exaggerated or understated. If the patient was <18 years of age or depending on the patient’s degree of neurodevelopmental disorder, the guardian responded to the questionnaire. However, there may be limitations in reflecting the patient’s own opinions sufficiently. Future research using research tools that can reflect the opinions of children, adolescents, and patients with neurodevelopmental disorders is needed. In future research, not only the process of diagnosis but also the genetic counseling service provided by the physicians for patients with rare diseases should be investigated. Further, the awareness and satisfaction of the patients regarding the genetic counseling service should also be assessed.

In conclusion, an AI-based diagnostic program can facilitate the diagnostic process of rare pediatric genetic diseases. Clinicians, patients, and guardians are generally satisfied with the AI-based diagnostic program. As the number of AI-based health care solutions will increase in the medical genetics field, genetic counseling will be considered more important. Further study is required to evaluate the utility of these programs in various genetic diseases.

## Acknowledgments

We deeply appreciate the patients and their families for participating in this study.

## Author contributions

Conceptualization: In Hee Choi, Beom Hee Lee, Baik-Lin Eun

Data curation: Go Hun Seo, Beom Hee Lee, Baik-Lin Eun

Investigation: Go Hun Seo, Yoon-Myung Kim, Chong Kun Cheon, Yoo-Mi Kim, Arum Oh, Jung Hye Byeon, Eungu Kang, Young-Lim Shin, Ji Eun Lee, Su Jin Kim, Hee Joon Yu, Woo Jin Kim, Byung Yoon Choi, Bong Jik Kim, Young Ho Kim, Gi Jung Im, Hyo-Jeong Lee, Hyun Ji Kim, Se-Hee Han

Methodology: In Hee Choi, JeongYun Park

Supervision: Beom Hee Lee, Baik-Lin Eun

Validation: Beom Hee Lee, Baik-Lin Eun

Visualization: In Hee Choi, JeongYun Park

Writing – original draft: In Hee Choi, Beom Hee Lee, Baik-Lin Eun

Writing – review & editing: In Hee Choi, Beom Hee Lee, Baik-Lin Eun
